# The Effect of Rosuvastatin in a Murine Model of Influenza A Infection

**DOI:** 10.1371/journal.pone.0035788

**Published:** 2012-04-20

**Authors:** Kathryn A. Radigan, Daniela Urich, Alexander V. Misharin, Sergio E. Chiarella, Saul Soberanes, Angel Gonzalez, Harris Perlman, Richard G. Wunderink, G. R. Scott Budinger, Gökhan M. Mutlu

**Affiliations:** 1 Division of Pulmonary and Critical Care Medicine, Northwestern University Feinberg School of Medicine, Chicago, Illinois, United States of America; 2 Division of Rheumatology, Northwestern University Feinberg School of Medicine, Chicago, Illinois, United States of America; 3 Department of Cell and Molecular Biology, Northwestern University Feinberg School of Medicine, Chicago, Illinois, United States of America; University of Liverpool, United Kingdom

## Abstract

**Rationale:**

HMG-CoA reductase inhibitors such as rosuvastatin may have immunomodulatory and anti-inflammatory effects that may reduce the severity of influenza A infection. We hypothesized that rosuvastatin would decrease viral replication, attenuate lung injury, and improve mortality following influenza A infection in mice.

**Methods:**

C57Bl/6 mice were treated daily with rosuvastatin (10 mg/kg/day) supplemented in chow (or control chow) beginning three days prior to infection with either A//Udorn/72 [H3N2] or A/WSN/33 [H1N1] influenza A virus (1×10^5^ pfu/mouse). Plaque assays were used to examine the effect of rosuvastatin on viral replication *in vitro* and in the lungs of infected mice. We measured cell count with differential, protein and cytokines in the bronchoalveolar lavage (BAL) fluid, histologic evidence of lung injury, and wet-to-dry ratio on Day 1, 2, 4, and 6. We also recorded daily weights and mortality.

**Results:**

The administration of rosuvastatin had no effect on viral clearance of influenza A after infection. Weight loss, lung inflammation and lung injury severity were similar in the rosuvastatin and control treated mice. In the mice infected with influenza A (A/WSN/33), mortality was unaffected by treatment with rosuvastatin.

**Conclusions:**

Statins did not alter the replication of influenza A *in vitro* or enhance its clearance from the lung *in vivo*. Statins neither attenuated the severity of influenza A-induced lung injury nor had an effect on influenza A-related mortality. Our data suggest that the association between HMG CoA reductase inhibitors and improved outcomes in patients with sepsis and pneumonia are not attributable to their effects on influenza A infection.

## Introduction

Influenza is the leading cause of death from an infectious cause and ranks 8^th^ in the list of attributable annual mortality in the United States [1]. During the pandemic of 1918–1919, influenza A virus caused as many as 50–100 million deaths [2]. In 2009, the World Health Association (WHO) recognized a pandemic infection with a newly identified strain of the influenza A virus (H1N1). The Centers for Disease Control and Prevention (CDC) estimated that 61 million cases, 274,000 hospitalizations, and 12,470 deaths were attributable to this strain in the United States from April 2009 to April 2010 [3]. Fedson and Dunnill estimated that 85% of developing countries were without vaccinations or treatment during the recent pandemic [4].

Even if a vaccine is effective and the expected challenges in generating an adequate supply are overcome, a substantial percentage of the population will develop an influenza infection during the next pandemic. Antiviral treatments are available but these treatments are expensive, limited and may become ineffective as resistant strains emerge [5], [6]. For example, during one of the most recent outbreaks in the United States, 185 of 190 H1N1 influenza virus isolates from 30 states were found to be resistant to oseltamivir and investigators in Australia have recently observed the emergence of oseltamivir resistant novel H1N1 strains [7], [8].

In light of these concerns, alternative treatments that are readily available and affordable are urgently needed. Inhibitors of HMG-CoA reductase (or “statins”) catalyze the conversion of hydroxymethylglutaryl-CoA to mevalonate and are widely used for treatment of hyperlipidemia. In addition to their cholesterol lowering effects, statins exert both anti-inflammatory and immunomodulatory properties [9]. These effects are thought to result from downstream effects of HMG-CoA reductase inhibition on isoprenoid lipid production which may alter G-protein signaling, cell proliferation, and the activity of adhesion molecules [10]. In support of this hypothesis, several groups of investigators have reported that statins significantly reduce mortality in observational studies conducted in patients with sepsis and bacterial pneumonia [11]–[20]. Rosuvastatin is one of the most commonly prescribed HMG-CoA reductase inhibitors. We sought to test the hypothesis that the administration of rosuvastatin to mice would alter influenza A viral clearance or the clinical course of influenza infection.

## Methods

### Ethics Statement

The protocol for the use of mice (ASP-2009-1041 and ASP-2010-1938) was approved by the Animal Care and Use Committee at Northwestern University.

### Influenza A virus and cells

Influenza A virus (A//Udorn/72 [H3N2]) and (A/WSN/33 [H1N1]) were provided by Robert Lamb, Ph.D., Sc.D., Northwestern University, Evanston, IL. Madin-Darby Canine Kidney (MDCK) cells (American Type Culture Collection (ATCC), Manassas, VA) cells were maintained in Dulbecco's modified Eagle's medium (DMEM) supplemented with 1% penicillin G/streptomycin and 10% fetal bovine serum (37°C, 5% CO_2_).

### Viral plaque assay

We grew MDCK cells in 6-well plates to 70–90% confluency and then treated them with DMEM supplemented with rosuvastatin (5 µM) for 24 hours. Once the cells reached 100% confluency, we incubated the cells with serial 10-fold dilutions of influenza A virus (10^1^–10^6^ pfu) in DMEM and 1% bovine serum albumin (BSA) at 37°C and aspirated the inoculums 1 hour later [21]. We added 3 ml of replacement media [2.4% Avicel (FMC BioPolymer, Philadelphia, PA), 2× DMEM, and N-acetyl trypsin (1.5 µg)] to each well and incubated them for 3 days [22]. We then removed the overlay and visualized viral plaques using naphthalene black dye solution (0.1% naphthalene black, 6% glacial acetic acid, 1.36% anhydrous sodium acetate) [21], [22].

### Animals and administration of influenza A virus and rosuvastatin

We used male C57BL/6 mice (8–12 weeks of age, 20–25 g) from Charles River. We obtained rosuvastatin supplemented chow formulations (485 mg/kg diet) from Harlan Laboratories. The concentration of rosuvastatin in the chow was based on measurements of daily chow intake in mice before and after influenza A infection (3 and 1 g/day, respectively). The concentration of chow was designed to provide 10 mg/kg/day of rosuvastatin, which has been reported to achieve levels in mice similar to those observed in humans and improve cardiovascular endpoints in mice [23]–[25]. Three days after beginning rosuvastatin supplementation, we anesthetized the mice with isoflurane and intubated them using a 20 gauge angiocath [26]. We then instilled 1×10^5^ pfu/ml of influenza A virus (A//Udorn/72 [H3N2]) or (A/WSN/33 [H1N1]) in 50 µL of PBS through the catheter. We treated control mice with the same volume PBS. To compensate for the decreased oral intake in the infected mice, the influenza A infected mice were fed with a chow containing a 3-fold higher concentration of rosuvastatin beginning 24 hours after infection. Day 0 was defined as the day of infection. We weighed unanesthetized mice on a portable balance (Mettler-Toledo Inc., Columbus, OH) daily.

### Collection of bronchoalveolar lavage (BAL) fluid and measurement of total protein, and cytokines

We sutured a 20-gauge angiocath into the trachea via a tracheostomy and slowly infused and aspirated 0.8 mL of PBS three times, which was immediately placed on ice. We then centrifuged a 100 µL of the BAL fluid in a cytospin (1200 rpm for 5 minutes) and Wright stained the glass slides. We measured cell count using a hemocytometer (trypan blue (0.4%) and subsequently counted the differential after staining was completed. We centrifuged the remaining BAL fluid (200× *g*, 5 min) and measured BAL protein (Bradford) and cytokines (BD Cytometric Bead Array Mouse Inflammation Kit, San Diego, CA) in the supernatant [26].

### Characterization of lung inflammatory cell populations using flow cytometry

Mice were euthanized with Euthasol followed by a rapid thoracotomy. The right ventricle was cannulated and while the right atrium was clamped with forceps, 5 ml of PBS was infused. The lungs were removed *en bloc* and the large airways were dissected from the peripheral lung tissue. The latter was minced and homogenized using Lung Dissociation kit (Miltenyl Biotek, Auburn, CA, Catalogue #130-095-927) according to the manufacturer's instructions for 30 minutes at 37°C and passed through 70 µm nylon mesh to obtain a single cell suspension. Remaining red blood cells were lysed using BD Pharm Lyse (BD Biosciences, San Jose, CA). Cells were counted using Countess automated cell counter (Invitrogen, Carlsbad, CA); dead cells were excluded using trypan blue.

After Live/Dead staining with Aqua dye (Invitrogen, Carlsbad, CA), cells were incubated with FcBlock (BD Biosciences, San Jose, CA) and stained with mixture of fluorochrome conjugated antibodies (see Table S1 for the list of antibodies, clones, fluorochromes and manufacturers). Data were acquired on BD LSR II flow cytometer (BD Biosciences, San Jose, CA) (see Table S2 for instrument configuration). Compensation and data analysis were performed using FlowJo software (TreeStar, Ashland, OR). After gating out cell aggregates, debris and dead cells, immune cells were identified using the pan-hematopoietic marker CD45. Specific cell types were identified as follows: alveolar macrophages (highly autofluorescent and CD11c^hi^CD11b^int^), interstitial macrophages (CD11b^+^CD11c^+^MHC II^+/−^), monocytes (CD11b^+^CD11c^−^Ly6C^+^MHC II^+/−^), neutrophils (CD11b^+^Ly6G^+^), B cells (CD19^+^), CD4 T cells (CD4^+^), CD8 T cells (CD8^+^), NK cells (NK1.1^+^), CD103^+^ dendritic cells (CD11c^+^CD11b^int^MHC II^+^CD103^+^), CD103^−^ dendritic cells (CD11c^+^CD11b^+^MHC II^+^CD103^−^). Data presented as percent of cells in CD45^+^ gate. Expression of the activation markers presented as median fluorescence intensity (MFI).

### Preparation of lung homogenates for viral plaque assay

We perfused the right ventricle *in situ* with sterile PBS (>1 ml, until the lungs were clear). The lungs were removed and kept on ice prior to and during homogenization (Tissue Tearor, 30 s) in a flow cytometry tube with 1 ml of PBS. An additional 2 mL of PBS was added to the resulting homogenate and subjected to Dounce homogenization (20 strokes). The lung homogenate was centrifuged (4°C, 2000 rpm for 10 minutes). We then incubated the cells with serial 10-fold dilutions of the supernatant in DMEM and 1% bovine serum albumin (BSA) at 37°C and aspirated the inoculums 1 hour later [21]. We measured the viral titer in lung homogenates as described above.

### Assessment of lung histopathology and lung water content

We perfused the right ventricle *in situ* with sterile PBS (1 ml) and then sutured a 20-gauge angiocath into the trachea via a tracheostomy. We then removed the lungs *en bloc* and inflated them to exactly 15 cm of H_2_O with 4% paraformaldehyde. We examined 5 µm sections from paraffin embedded lungs stained with hematoxylin-eosin using light microscopy. We evaluated the lung water (edema) content by calculating the ratio of wet-to-dry lung weights. Both left and right lungs were weighed before and after oven desiccation (>72 hours) to calculate wet-to-dry lung ratio.

### Mortality

We continuously observed mice infected with influenza A virus (A/WSN/33 [H1N1]) for signs of distress (slowed respiration, failure to respond to cage tapping, failure of grooming and fur ruffling). Mice that developed these symptoms were sacrificed and the death was recorded as an influenza A-induced mortality. The majority of the mice died without developing these signs, when this occurred, the death was recorded as mortality.

### Statistics

We explored differences between groups using analysis of variance (ANOVA). When the ANOVA indicated a significant difference, we explored individual differences using *t* tests with a Dunnett or Tukey's correction for multiple comparisons as indicated. We performed all analyses using GraphPad Prism version 4.00 for Windows (GraphPad Software, San Diego CA, USA). Data are shown as means ± SE. A *p* value<0.05 was considered statistically significant for all tests.

## Results

### Rosuvastatin does not inhibit influenza A viral replication *in vitro* or *in vivo*

**Figure 1:**
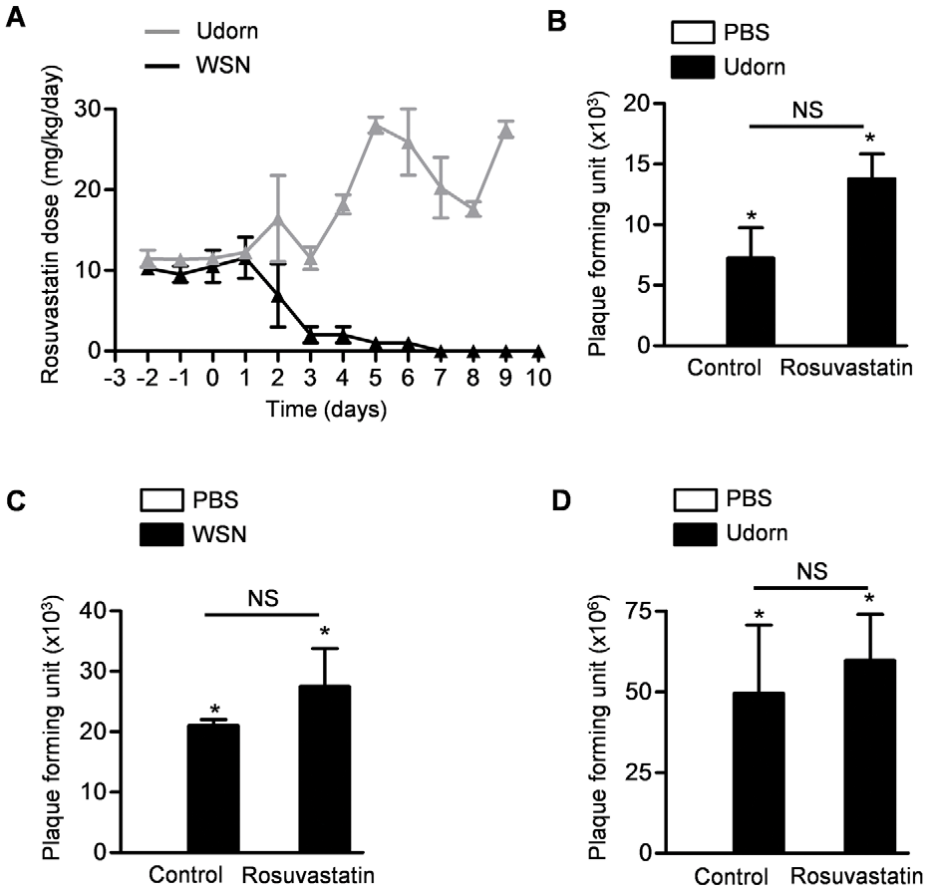
Rosuvastatin does not inhibit influenza A viral replication *in vitro* or *in vivo*. (**A**) The oral intake of rosuvastatin by mice infected with either Udorn or WSN strains of influenza A virus over time. We treated mice with rosuvastatin or control therapy starting 3 days before they were infected with either Udorn or WSN strains of influenza A virus. Four days after influenza A infection, we measured viral titers (plaque forming unit [pfu]) in lung homogenates harvested from mice treated with either (B) Udorn or (C) WSN strain. (**D**) Plaque forming units were counted in MDCK cells treated with rosuvastatin (5 µM) beginning 24 hours prior to infection with influenza A virus (Udorn) *in vitro*. *P<0.05 Udorn vs. PBS, WSN vs. PBS. NS; not significant (Rosuvastatin vs. Control treatment).

To determine the appropriate concentrations of rosuvastatin in the chow, we measured food intake in mice before and after infection with Udorn or WSN strains of influenza A virus. Mice treated with influenza A Udorn maintained a constant intake of rosuvastatin at about 1/3 of the untreated value throughout the duration of the experiment. Mice infected with influenza A WSN stopped eating 2 days after infection. We used these values to determine rosuvastatin intake in mice infected with either the two strains of influenza A virus (Fig. 1A).

We infected mice with influenza A Udorn and measured viral titers in lung homogenates obtained in the first 6 days after infection. Viral titers were highest one day after infection and declined thereafter (Fig. S1). We infected mice that had been treated for 3 days with rosuvastatin supplemented chow with influenza A virus and collected lung homogenates 1, 2 and 4 days later (Udorn) or 1 and 4 days later (WSN). There was no significant difference in viral titers between the control and rosuvastatin treated mice at any time point (Fig. 1B, Udorn; Fig. 1C, WSN; only the day 4 value is shown; others are shown in Figure S2). To determine whether rosuvastatin reduces influenza A viral replication *in vitro*, we treated 70% confluent MDCK cells with rosuvastatin in media or media alone (control) 24 hours before the cells were infected with influenza A virus (Udorn) and measured viral replication using plaque assays 72 hours later. We observed no difference in viral replication between the control-and rosuvastatin-treated cells (Fig. 1D).

### Rosuvastatin does not alter the clinical course of influenza A infection in mice

**Figure 2:**
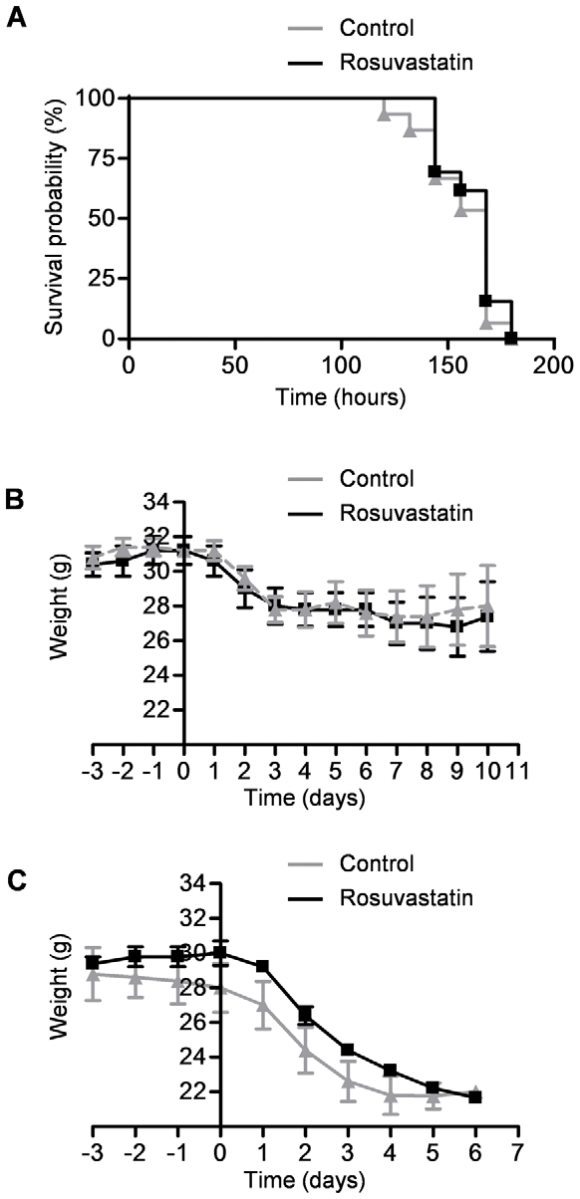
Rosuvastatin does not alter the clinical course of influenza A infection in mice. We treated mice with rosuvastatin or control therapy starting 3 days before they were infected with either Udorn or WSN strains of influenza A virus and measured (**A**) WSN-associated mortality and (**B**) Udorn- and (**C**) WSN-associated changes in daily weight.

We treated mice with rosuvastatin prior to and following infection with influenza A WSN as described above and followed them for up to 14 days after infection and recorded mortality. There were no significant differences in mortality between the rosuvastatin or control treated mice (Fig. 2A). We then measured weight loss in mice treated with rosuvastatin or control in the 10 days after infection with either Udorn or WSN strains of influenza A virus. Infection with either strain of influenza A was associated with the loss of approximately 30% of body weight. There was no difference in influenza A-induced weight loss or recovery between the rosuvastatin and control treated mice (Fig. 2B, Udorn; Fig. 2C, WSN, mortality in the WSN treated mice precluded measurements beyond 7 days).

### Rosuvastatin does not have an effect on influenza A-induced lung inflammation

**Figure 3:**
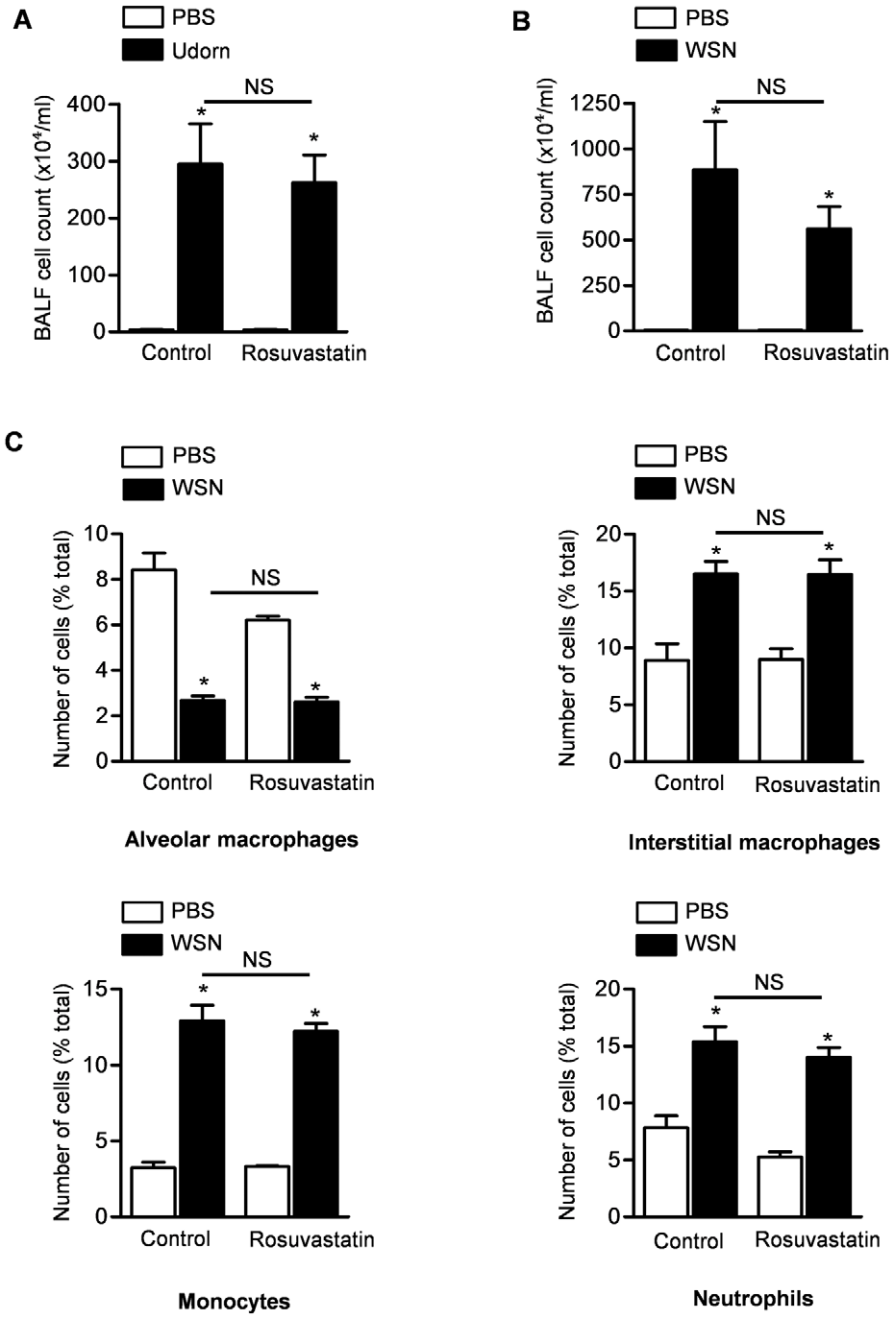
Rosuvastatin does not have an effect on influenza A-induced changes in inflammatory cell count and differential in the lungs. We treated mice with rosuvastatin or control therapy starting 3 days before they were infected with either Udorn or WSN strains of influenza A virus. Four days after influenza A infection, (**A**, **B**) we measured cell count in the bronchoalveolar lavage fluid (BALF). (**C**) We also performed flow cytometry in digested lung tissue from mice treated with WSN strain of influenza A virus to determine differential count of inflammatory cells including alveolar and interstitial macrophages, monocytes and neutrophils. *P<0.05 Udorn vs. PBS, WSN vs. PBS. NS; not significant (Rosuvastatin vs. Control treatment).

We measured BAL fluid cell count in mice 4 days after infection of mice with either Udorn or WSN strains of influenza A virus. Similar to reports from other groups, we observed an increase in BAL fluid cellularity (predominantly neutrophils and macrophages) following infection with either strain of influenza virus. Total leukocyte counts in the BAL fluid were similar in both the rosuvastatin- and control-treated mice (Fig. 3A, Udorn; Fig. 3B, WSN; other time points after infection shown in Figure S3A). We then performed selected immunophenotyping of the inflammatory cell populations (CD45^+^) in lung digests. Infection with influenza A caused a reduction in the relative percentage of alveolar macrophages and an increase in the percentage of interstitial macrophages, blood derived monocytes and neutrophils in the lung (Fig. 3C). Influenza A infection also caused a reduction in lymphocytes, an increase in monocyte-derived dendritic cells and no significant change in natural killer cells or CD103^+^ dendritic cells in the lungs (Fig. S3B). Rosuvastatin treatment did not have an effect on influenza A-induced changes in the percentage of inflammatory cells (Fig. 3C and Fig. S3B).

**Figure 4:**
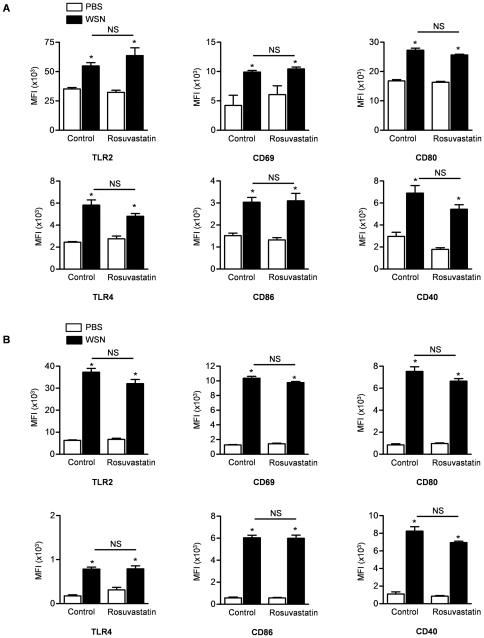
Rosuvastatin does not alter the influenza A-induced expression of activation markers on inflammatory cells in the lungs. We treated mice with rosuvastatin or control therapy starting 3 days before they were infected with WSN strains of influenza A virus. Four days after influenza A infection, we performed flow cytometry in digested lung tissue to determine the effect of rosuvastatin on activation markers expressed on (**A**) alveolar macrophages and (**B**) interstitial macrophages. *P<0.05 WSN vs. PBS. NS; not significant (Rosuvastatin vs. Control treatment).

Using flow cytometry, we also looked at expression of the activation markers TLR4, TLR2, CD40, CD69, CD80, and CD86 on alveolar macrophages (Fig. 4A), interstitial macrophages (Fig. 4B), and monocyte-derived (CD103^−^) dendritic cells (Fig. S4). While influenza A infection was associated with increased expression of all of these markers, there were no significant differences between mice treated with rosuvastatin compared with mice treated with control chow.

**Figure 5:**
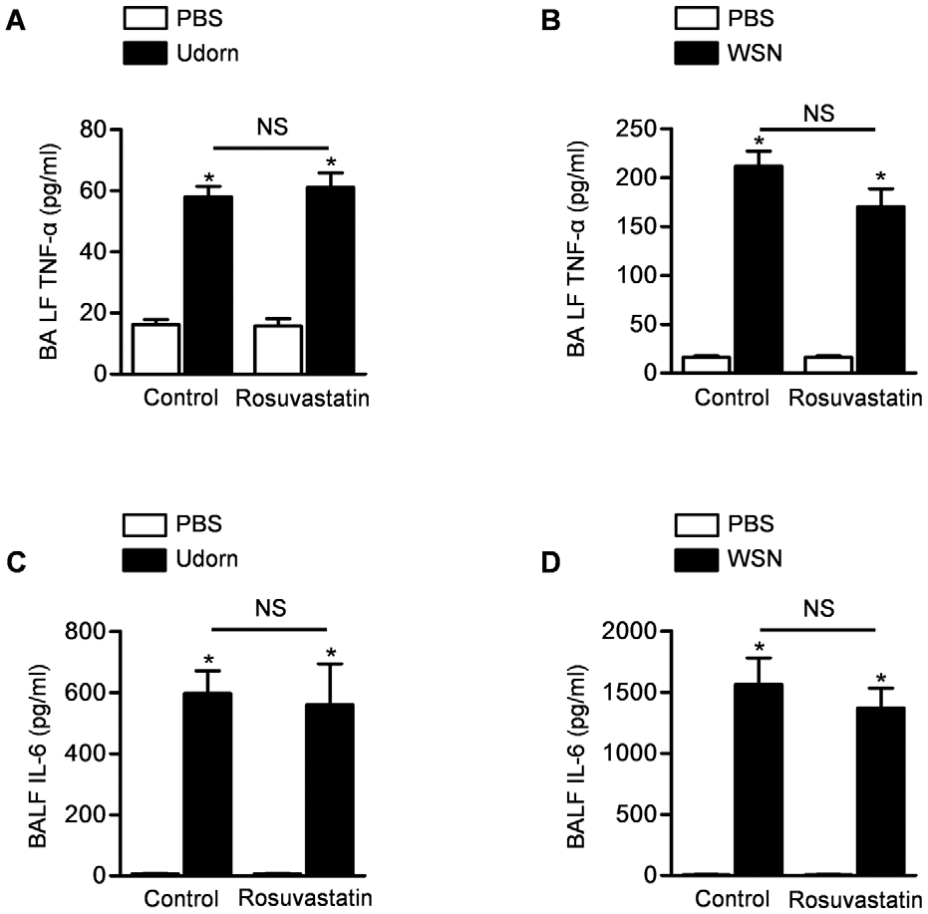
Rosuvastatin does not affect the influenza A-induced changes in pro-inflammatory cytokines in the lungs. We treated mice with rosuvastatin or control starting 3 days before they were infected with either Udorn or WSN strains of influenza A virus. Four days after influenza A infection, we collected bronchoalveolar lavage fluid (BALF) and measured (**A, B**) TNF-α and (**C, D**) IL-6 levels. *P<0.05 Udorn vs. PBS, WSN vs. PBS. NS; not significant (Rosuvastatin vs. Control treatment).

We then used a multiplex proinflammatory cytokine bead array to measure the levels of pro-inflammatory cytokines in the BAL fluid of mice infected 4 days earlier with either Udorn or WSN strain of influenza A virus. Similar to reports from other investigators, infection with influenza A was associated with an increase in BAL fluid pro-inflammatory cytokines including TNF-α (Fig. 5A, Udorn; Fig. 5B, WSN; other time points after infection shown in Figure S5A) and IL-6 (Fig. 5C, Udorn; Fig. 5D, WSN; other time points after infection shown in Figure S5B); however, there were no significant differences in any of the cytokines measured between control and rosuvastatin-treated mice (Fig. 5; other cytokines are shown in Figure S5C).

### Rosuvastatin does not modify the severity of influenza A-induced lung injury

**Figure 6:**
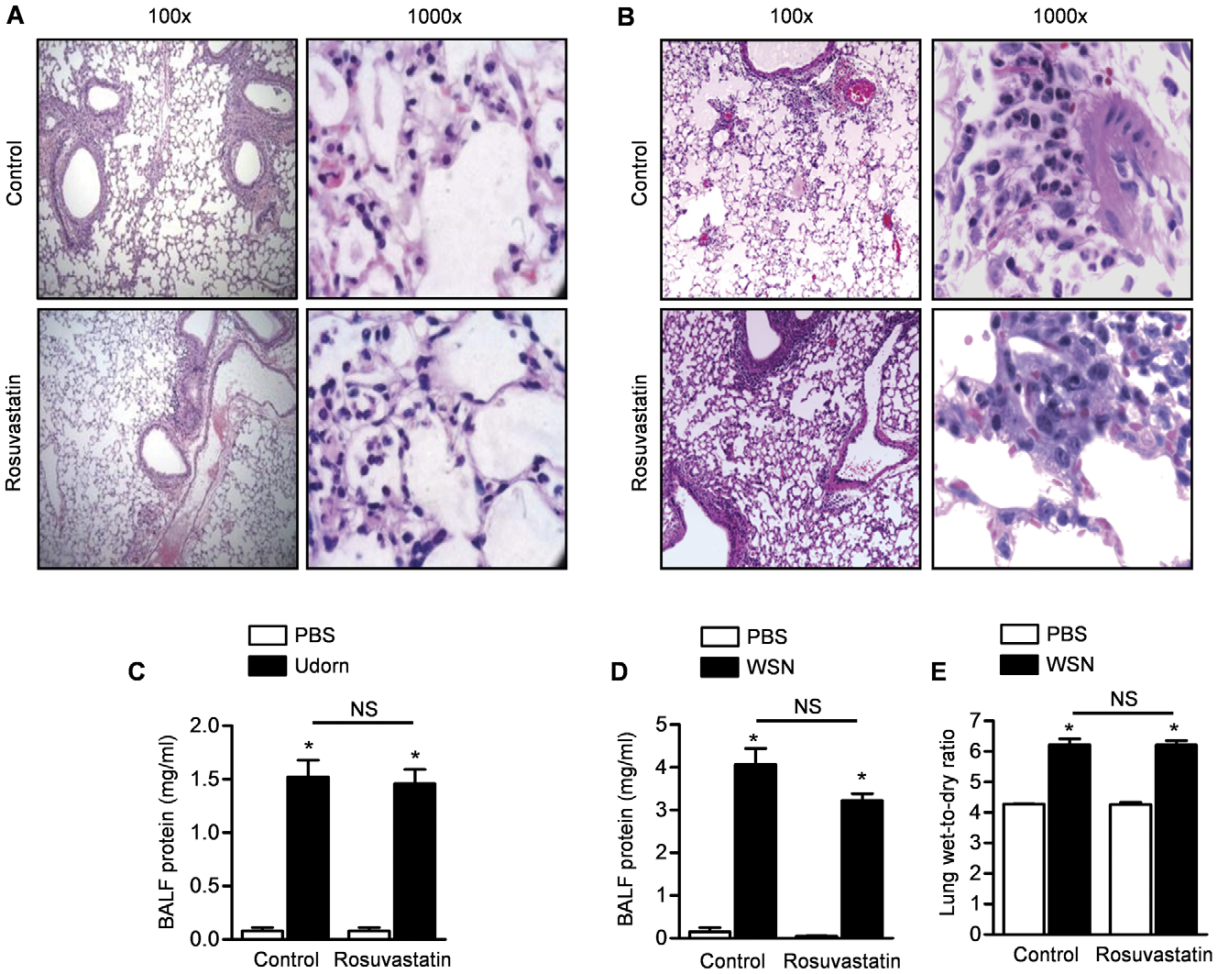
Rosuvastatin does not modify the severity of influenza A-induced lung injury. We treated mice with rosuvastatin or control starting 3 days before they were infected with either Udorn or WSN strains of influenza A virus. (**A, B**) Four days after influenza A infection, we collected lungs for assessment of lung histology. Representative low (×100) and high (×1,000) magnification images of lung sections from mice treated with either the (**A**) Udorn or (**B**) WSN strains. (**C, D**) BALF protein concentrations in mice 4 days after infection with either the (**C**) Udorn or (**D**) WSN strains. (**E**) Wet-to-dry weight ratio of lungs from mice 6 days after infection with the WSN strain. *P<0.05 Udorn vs. PBS, WSN vs. PBS. NS; not significant (Rosuvastatin vs. Control treatment).

We treated mice with rosuvastatin beginning 3 days before infection with the influenza A WSN virus and sacrificed the mice for examination of hematoxylin and eosin stained lung sections on Day 4 after infection. Compared with saline treated mice, we observed an increase in interstitial inflammatory cells, lung edema and hemorrhage in the mice infected with either the influenza A Udorn or WSN strains. However, there were no significant differences in the histologic severity of the lung injury between the influenza A-infected mice treated with rosuvastatin or control chow (Fig. 6A, Udorn; Fig. 6B, WSN). Compared with control-treated mice, we observed an increase in BAL fluid protein in influenza A Udorn- or WSN-infected mice after infection (Fig. S6). Again, there were no significant differences in BAL fluid protein between mice treated with rosuvastatin or control chow (Fig. 6C, Udorn; Fig. 6D, WSN; only Day 4 value is shown, others are shown in Figure S6). Compared with control treated mice, we observed an increase in wet-to-dry weight in mice treated with influenza A WSN but not Udorn (Fig. S6). We saw no difference in the increase in wet-to-dry weight ratio in the influenza A WSN-treated mice treated with rosuvastatin (Fig. 6E).

## Discussion

HMG-CoA reductase inhibitors are now the standard of care for treating hyperlipidemia. Since their adoption into widespread clinical use, several groups of investigators have reported apparent benefits of statins in observational studies of patients with sepsis and pneumonia, suggesting they may confer protection via a mechanism independent of their lipid lowering effects [11]–[16], [27]. In murine models of infection with two common strains of influenza A virus, we found that orally administered rosuvastatin did not alter influenza A viral replication in the lung, viral-mediated inflammation, the development of acute lung injury or mortality.

Several groups of investigators have suggested that some of the benefit from statins result from an inhibition of inflammation [28]–[32]. In normal volunteers, intranasal infection with influenza A virus results in the release of TNF-α followed rapidly by the release of IL-6 and other pro-inflammatory cytokines. In those patients, nasal lavage and plasma levels of IL-6 best correlated with influenza A symptoms [33]. In patients with a dilated cardiomyopathy, Node et al reported that the administration of statins was associated with a reduction in the levels of TNF-α and IL-6 [34]. We therefore reasoned that the administration of statins would alter the clinical course of influenza A infection by reducing the levels of these cytokines. However, we observed that the influenza-A-induced increase in pro-inflammatory cytokines was similar in mice treated with rosuvastatin and control treated mice.

Investigators have reported an inverse association between statin use and poor clinical outcomes in observational studies of patients with sepsis, community-acquired pneumonia (CAP), bacteremia without sepsis, and in post-operative patients. Furthermore, a recent randomized, open-label, controlled trial showed a non-significant trend toward improved outcomes in critically ill patients receiving statins [35]. While these benefits may have resulted from the immunomodulatory or antimicrobial effects of statins against certain pathogens [30]–[32], our results suggest these effects would be minimal during influenza A infection. Alternatively, the improved outcomes might result from the well-established beneficial effects on statins on cardiovascular outcomes. In support of this hypothesis, ischemic cardiovascular complications are increasingly recognized as important contributors to pneumonia related morbidity and mortality [36]–[38]. Furthermore, in population based observational studies investigators have reported a positive association between influenza A infection and an acute increase in cardiovascular and cerebrovascular diseases, which drive a significant portion of the excess mortality [36]–[38].

Statins may function as antimicrobials against some organisms. For example the administration of statins has been shown to prevent respiratory syncytial viral replication *in vitro*, perhaps as a result of isoprenylation of RhoA, which binds to a viral fusion glycoprotein required for RSV syncytium formation the administration [39]. RhoA and its binding to viral fusion glycoprotein are necessary for RSV syncytium formation. Statins are known to inhibit the production of isoprenyl groups and as a result prevent its localization in the plasma membrane, limit entry, and attenuate replication of RSV. Since the replication of influenza A virus is different than RSV, it is not surprising that rosuvastatin had no effect on the replication rate of Influenza A virus *in vivo* or *in vitro*. In addition statins have been shown to inhibit the growth of salmonella, yeast and the human immunodeficiency virus *in vitro*; however, the *in vivo* relevance of these findings is unknown [40]–[43]. We found that rosuvastatin did not alter influenza A viral replication *in vitro* or change the rate of viral clearance *in vivo*.

We pretreated mice with rosuvastatin for 3 days prior to influenza A infection and maintained this dose throughout the infection in mice treated with Udorn strain. In these mice, we did not observe any difference in the clinical course of influenza in the control or rosuvastatin treated mice. However, in mice treated with the WSN virus, oral intake virtually ceased 1–2 days after the influenza infection and the mice became too ill to tolerate oral gavage, precluding maintenance of the same dose. As a result, we cannot exclude the possibility that a longer duration of pretreatment or that maintenance of the dose in the premorbid stages after infection with the WSN virus may have shown an effect. Nevertheless, our results do not support a role for rosuvastatin as a therapy in the early in the stages of influenza A infection. We also speculate that the effect of statin therapy post-infection would be negligible.

Weight loss is an important clinical component of influenza A infection. Pro-inflammatory cytokines, including TNF-α, interleukin (IL)-1β, and IL-6 have been implicated in the loss of appetite and weight in influenza infection, chronic inflammatory disorders and cancer [44]–[46]. In experimental models, reduced levels of inflammatory cytokines have been shown to attenuate influenza A-induced weight loss [46], [47].

We conclude that in two murine models of influenza A pneumonia, the administration of rosuvastatin did not alter the viral clearance of influenza from the lung. Furthermore, rosuvastatin did not attenuate the severity of influenza A-induced lung injury and did not have an effect on influenza A related mortality. Our results do not support the use of statins in patients exposed to or diagnosed with acute influenza A infection who lack other indications for their use.

## Supporting Information

Figure S1 **Influenza A viral titers in mouse lung tissue.** We infected mice with influenza A virus (Udorn) and measured viral titers in lung homogenates obtained in the first 6 days after infection.
(TIF)

Figure S2 **The effect of rosuvastatin on influenza A viral titers in mouse lung tissue.** We treated mice with rosuvastatin or control starting 3 days before they were infected with either Udorn or WSN strains of influenza A virus and measured (**A**) Udorn and (**B**) WSN viral titers (plaque forming unit) in lung homogenates on day 1 (D1), D2 and D4 after infection.
(TIF)

Figure S3 **The effect of rosuvastatin on influenza A-induced changes in inflammatory cell count and differential in the lungs.** We treated mice with rosuvastatin or control therapy starting 3 days before they were infected with either Udorn or WSN strains of influenza A virus and measured (**A**) Udorn- and (**B**) WSN-associated changes in the bronchoalveolar lavage fluid (BALF) cell count on day 1 (D1), D2, D4 and D6 after infection. (**C**) We also performed flow cytometry in digested lung tissue from mice treated with WSN strain of influenza A virus to determine differential count of inflammatory cells including lymphocytes, dendritic cells and natural killer cells on day 4. *P<0.05 WSN vs. PBS. NS; not significant (Rosuvastatin vs. Control treatment).
(TIF)

Figure S4 **Rosuvastatin does not alter the influenza A-induced expression of activation markers on monocyte-derived dendritic cells in the lungs.** We treated mice with rosuvastatin or control therapy starting 3 days before they were infected with WSN strains of influenza A virus. Four days after influenza A infection, we performed flow cytometry in digested lung tissue to determine the effect of rosuvastatin on activation markers expressed on monocyte-derived dendritic cells. *P<0.05 WSN vs. PBS. NS; not significant (Rosuvastatin vs. Control treatment).
(TIF)

Figure S5 **The effect of rosuvastatin on influenza A-induced changes in cytokines.** We treated mice with rosuvastatin or control starting 3 days before they were infected with either Udorn or WSN strains of influenza A virus and measured the bronchoalveolar lavage fluid (BALF) levels of (**A**) TNF-α, (**B**) IL-6 and (**C**) other cytokines including monocyte chemotactic protein-1 (MCP-1), IL-10 and interferon-gamma (IFN-γ) on day 1 (D1), D2, and D4 after infection.
(TIF)

Figure S6 **The effect of rosuvastatin on influenza A-induced lung injury.** We treated mice with rosuvastatin or control starting 3 days before they were infected with either Udorn or WSN strains of influenza A virus and measured (**A, B**) Udorn- and (**C, D**) WSN-associated changes in the (**A, C**) bronchoalveolar lavage fluid (BALF) protein levels and (**B, D**) lung weight-to-dry ratio on day 1 (D1), D2, D4 and D6 after infection.
(TIF)

Table S1 Antibodies used flow cytometric analysis.
(DOCX)

Table S2 Configuration of the BD LSR II instrument.
(DOCX)
