# Supplementary figures and images for: The Effect of Rosuvastatin in a Murine Model of Influenza A Infection
Source: PLoS One. 2012 Apr 20;7(4):e35788. doi: 10.1371/journal.pone.0035788 (PMC3335012; doi:10.1371/journal.pone.0035788)

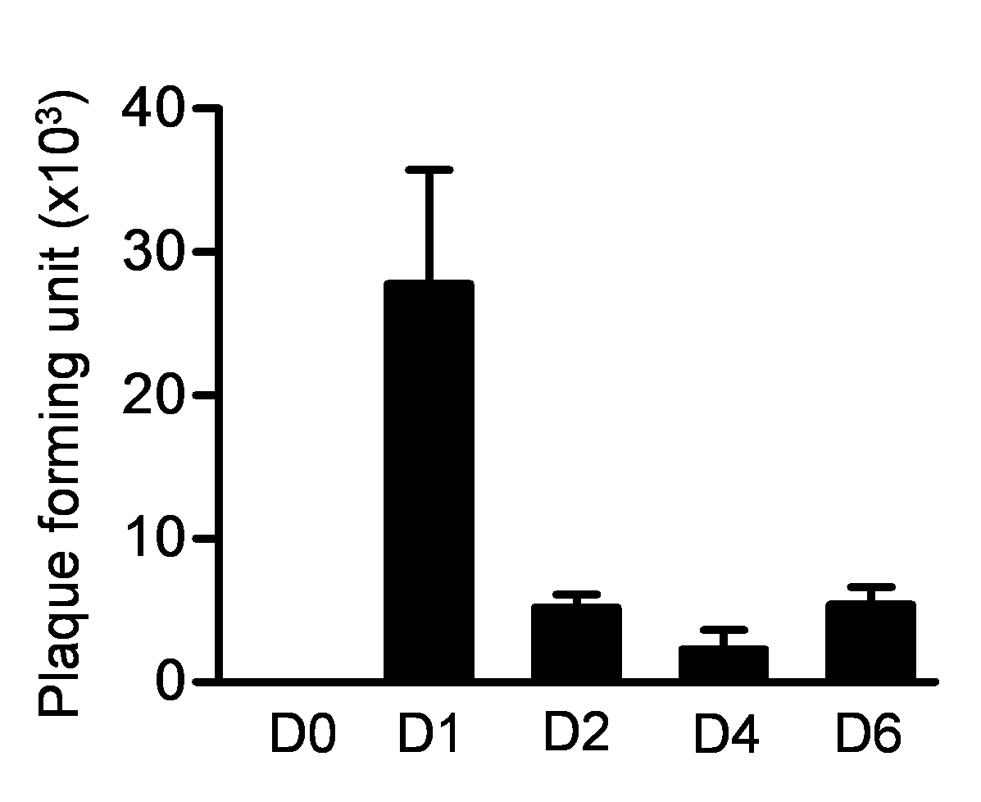

Supplement: Figure S1 — Influenza A viral titers in mouse lung tissue. We infected mice with influenza A virus (Udorn) and measured viral titers in lung homogenates obtained in the first 6 days after infection. (TIF) [file pone.0035788.s001.tif]

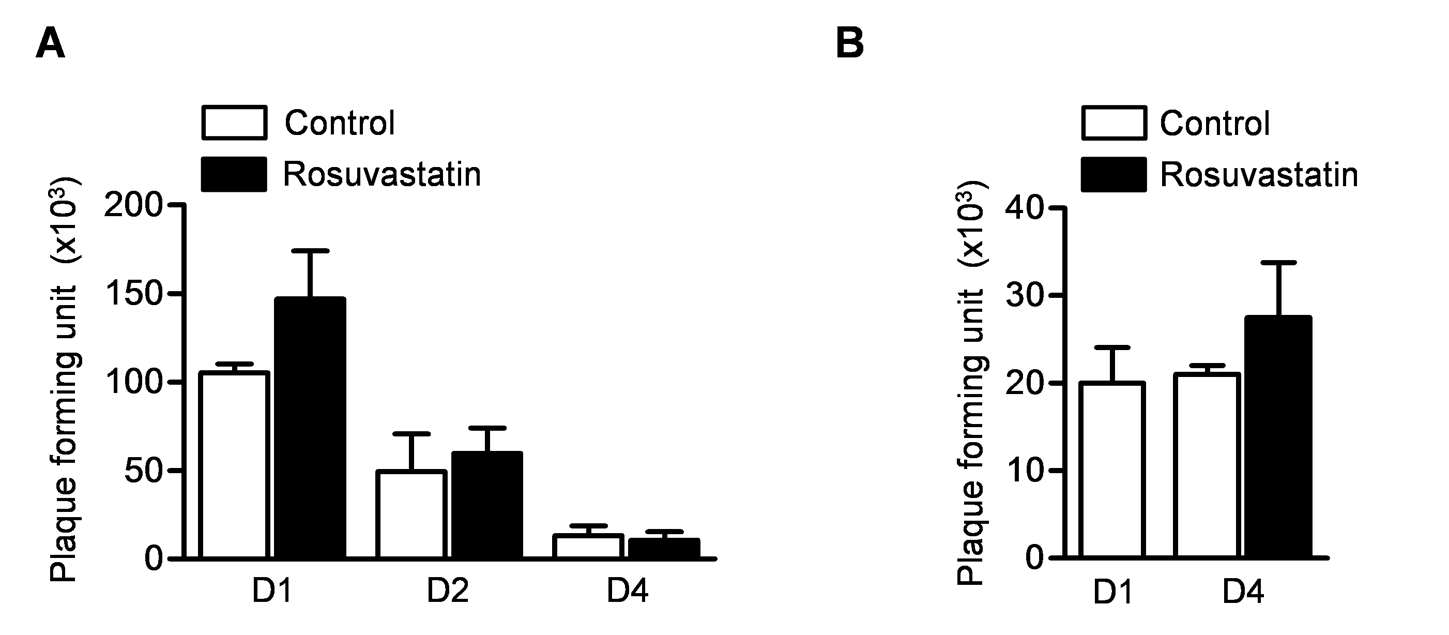

Supplement: Figure S2 — The effect of rosuvastatin on influenza A viral titers in mouse lung tissue. We treated mice with rosuvastatin or control starting 3 days before they were infected with either Udorn or WSN strains of influenza A virus and measured (A) Udorn and (B) WSN viral titers (plaque forming unit) in lung homogenates on day 1 (D1), D2 and D4 after infection. (TIF) [file pone.0035788.s002.tif]

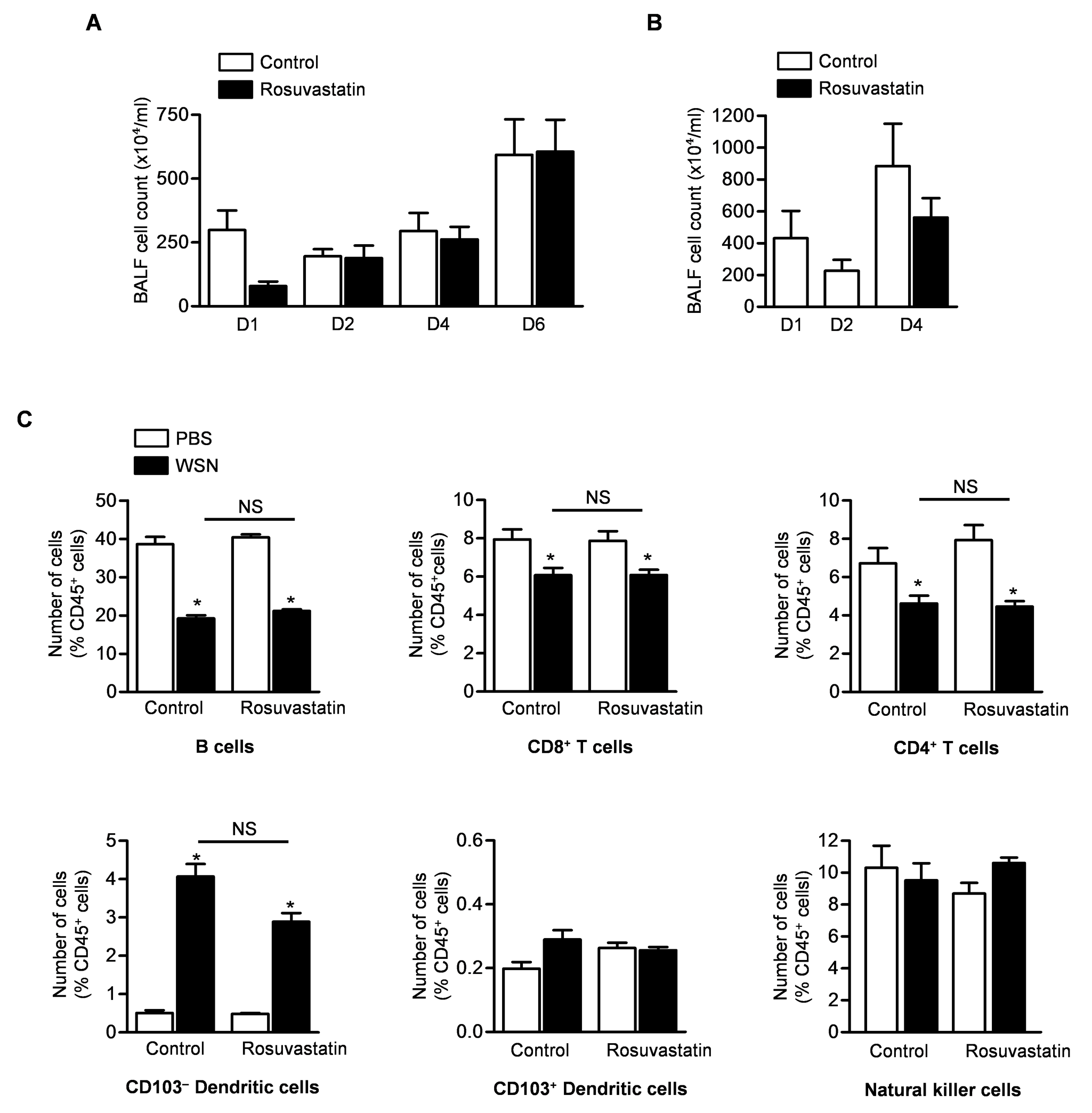

Supplement: Figure S3 — The effect of rosuvastatin on influenza A-induced changes in inflammatory cell count and differential in the lungs. We treated mice with rosuvastatin or control therapy starting 3 days before they were infected with either Udorn or WSN strains of influenza A virus and measured (A) Udorn- and (B) WSN-associated changes in the bronchoalveolar lavage fluid (BALF) cell count on day 1 (D1), D2, D4 and D6 after infection. (C) We also performed flow cytometry in digested lung tissue from mice treated with WSN strain of influenza A virus to determine differential count of inflammatory cells including lymphocytes, dendritic cells and natural killer cells on day 4. *P<0.05 WSN vs. PBS. NS; not significant (Rosuvastatin vs. Control treatment). (TIF) [file pone.0035788.s003.tif]

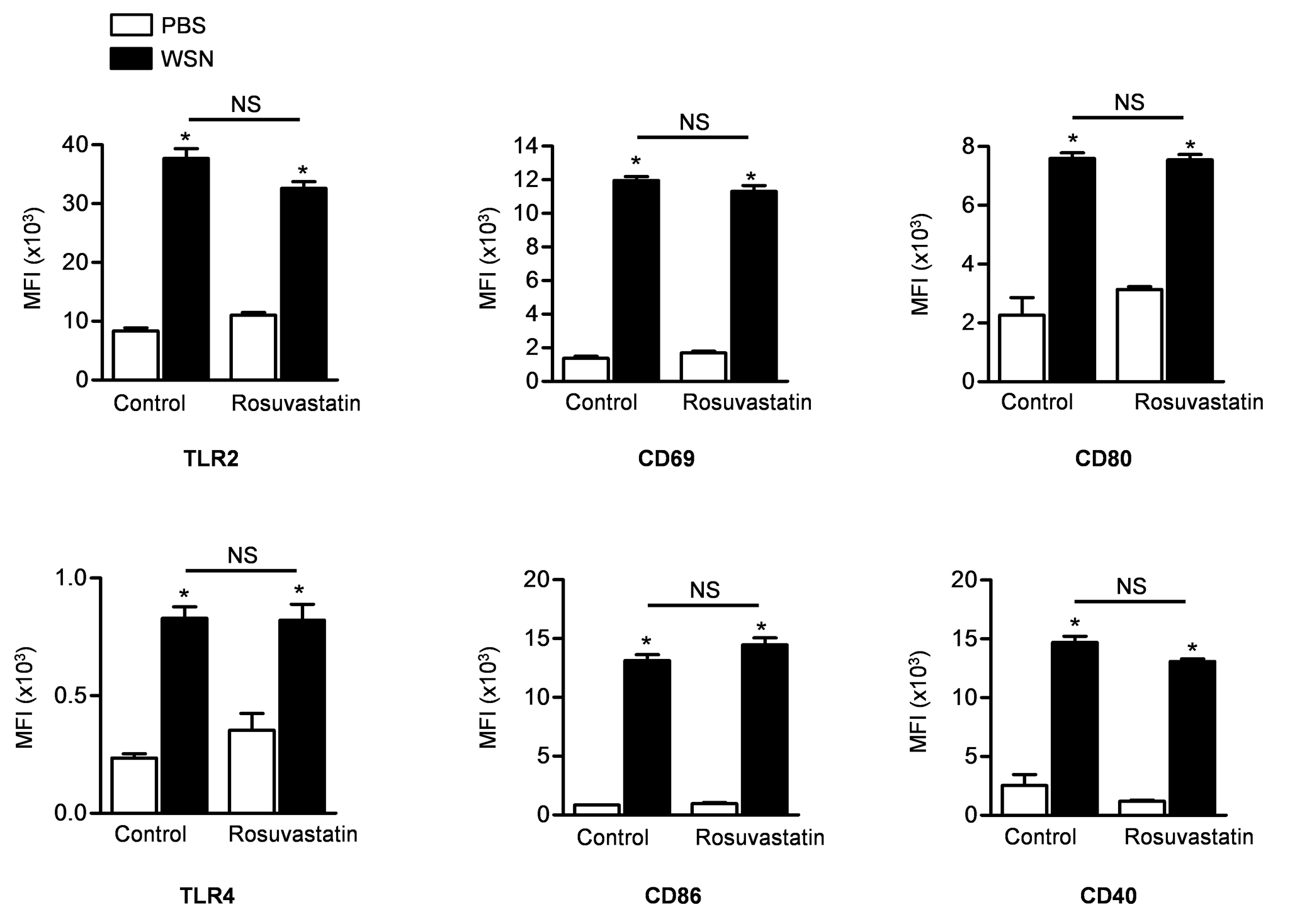

Supplement: Figure S4 — Rosuvastatin does not alter the influenza A-induced expression of activation markers on monocyte-derived dendritic cells in the lungs. We treated mice with rosuvastatin or control therapy starting 3 days before they were infected with WSN strains of influenza A virus. Four days after influenza A infection, we performed flow cytometry in digested lung tissue to determine the effect of rosuvastatin on activation markers expressed on monocyte-derived dendritic cells. *P<0.05 WSN vs. PBS. NS; not significant (Rosuvastatin vs. Control treatment). (TIF) [file pone.0035788.s004.tif]

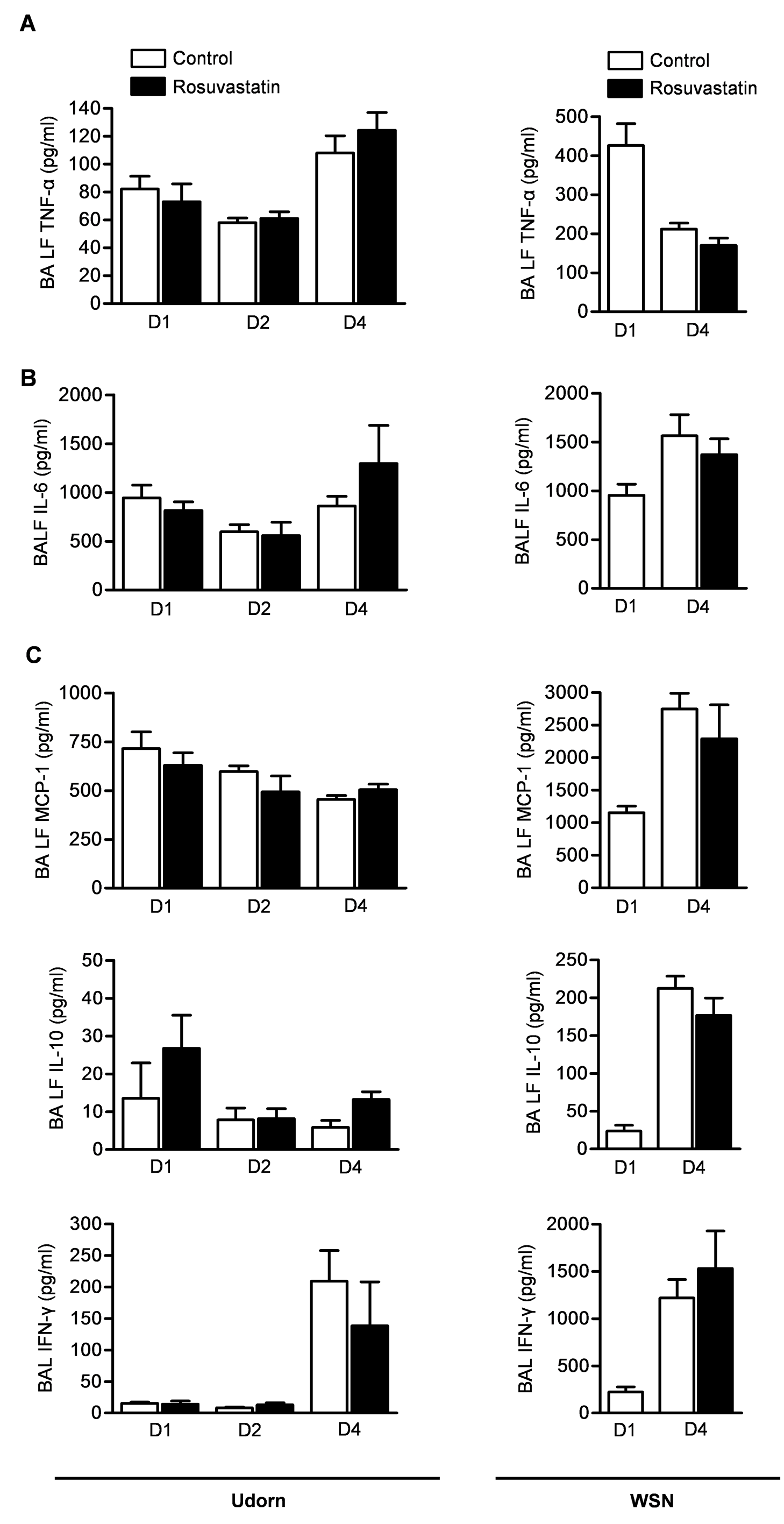

Supplement: Figure S5 — The effect of rosuvastatin on influenza A-induced changes in cytokines. We treated mice with rosuvastatin or control starting 3 days before they were infected with either Udorn or WSN strains of influenza A virus and measured the bronchoalveolar lavage fluid (BALF) levels of (A) TNF-α, (B) IL-6 and (C) other cytokines including monocyte chemotactic protein-1 (MCP-1), IL-10 and interferon-gamma (IFN-γ) on day 1 (D1), D2, and D4 after infection. (TIF) [file pone.0035788.s005.tif]

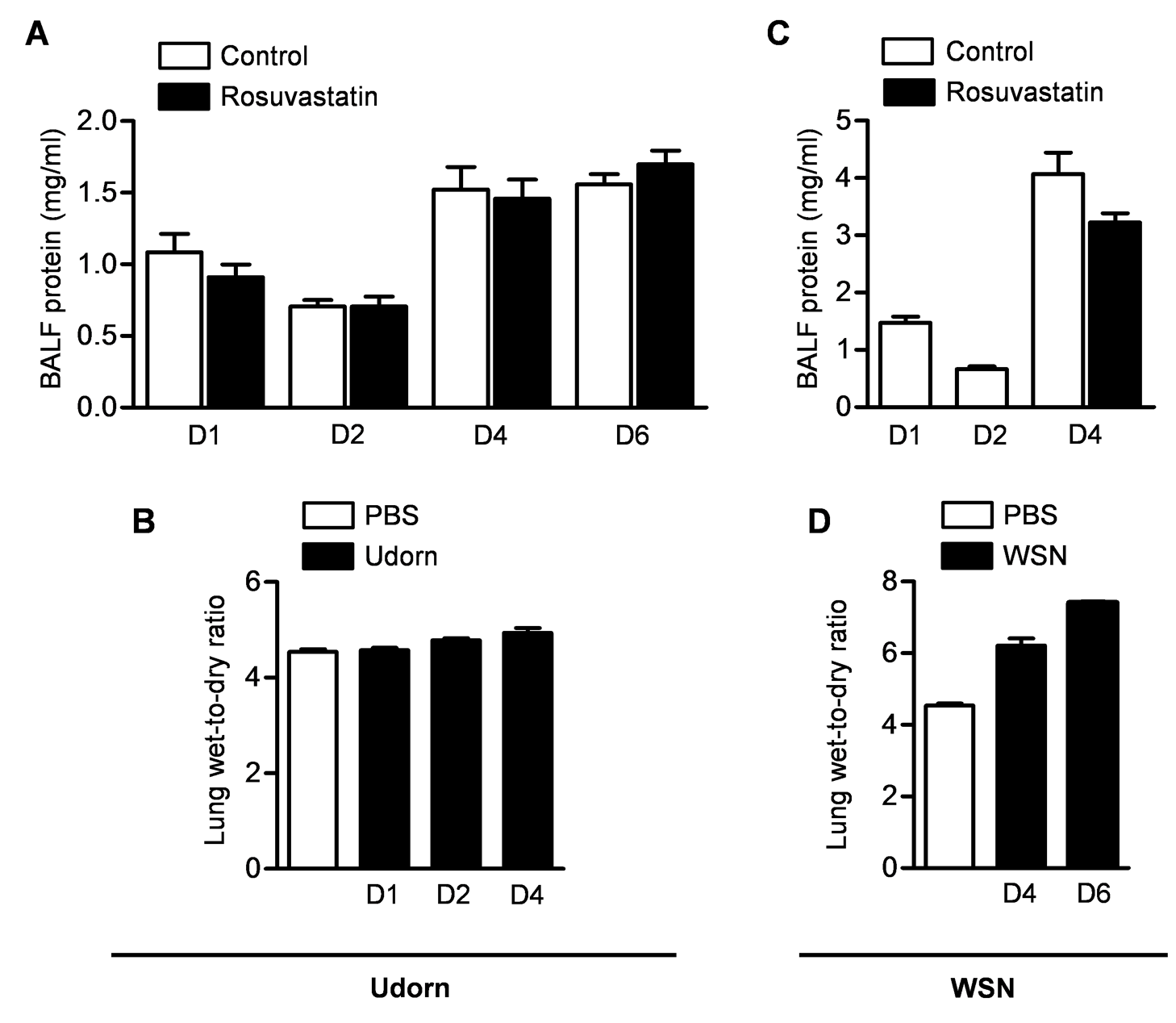

Supplement: Figure S6 — The effect of rosuvastatin on influenza A-induced lung injury. We treated mice with rosuvastatin or control starting 3 days before they were infected with either Udorn or WSN strains of influenza A virus and measured (A, B) Udorn- and (C, D) WSN-associated changes in the (A, C) bronchoalveolar lavage fluid (BALF) protein levels and (B, D) lung weight-to-dry ratio on day 1 (D1), D2, D4 and D6 after infection. (TIF) [file pone.0035788.s006.tif]
