# Supplementary material for: The Effect of Rosuvastatin in a Murine Model of Influenza A Infection
Source: PLoS One. 2012 Apr 20;7(4):e35788. doi: 10.1371/journal.pone.0035788 (PMC3335012; doi:10.1371/journal.pone.0035788)
Supplement: Table S1 — Antibodies used flow cytometric analysis. (DOCX) [file pone.0035788.s007.docx]

**Table S1. Antibodies used flow cytometric analysis**

| **Antigen** | **Clone** | **Fluorochrome** | **Manufacturer** |
| --- | --- | --- | --- |
| CD11b | M1/70 | PE-Texas Red | Invitrogen |
| CD11c | HL3 | APC  PE-Cy7 | BD Biosciences  BD Biosciences |
| CD19 | 1D3 | Alexa Fluor 700 | BD Biosciences |
| CD40 | HM40-3 | Alexa Fluor 647 | Biolegend |
| CD45 | 30-F11 | eFluor 450 | eBioscience |
| CD69 | H1.2F3 | PerCP-Cy5.5 | BD Biosciences |
| CD80 | 16-10A1 | PE | BD Biosciences |
| CD86 | GL1 | Alexa Fluor 700 | BD Biosciences |
| CD103 | 2E7 | FITC | eBioscience |
| F4/80 | BM8 | PE-Cy7 | eBioscience |
| Ly6G | 1A8 | PerCP-Cy5.5  Alexa Fluor 700 | BD Biosciences  BD Biosciences |
| Ly6C | AL-21 | APC-Cy7 | BD Biosciences |
| NK1.1 | PK136 | APC  Alexa Fluor 700 | BD Biosciences  BD Biosciences |
| MHC II (I-A/I-E) | M5/114.15.2 | PE  eFluor 450 | eBioscience  eBioscience |
| Siglec F | E50-2440 | PE | BD Biosciences |
| TLR2 | 6C2 | PE | eBioscience |
| TLR4 | MTS510 | PECy7 | eBioscience |
