# Supplementary material for: The Effect of Rosuvastatin in a Murine Model of Influenza A Infection
Source: PLoS One. 2012 Apr 20;7(4):e35788. doi: 10.1371/journal.pone.0035788 (PMC3335012; doi:10.1371/journal.pone.0035788)
Supplement: Table S2 — Configuration of the BD LSR II instrument. (DOCX) [file pone.0035788.s008.docx]

**Table S2. Configuration of the BD LSR II instrument**

| **Laser** | **Detector** | **Filter** | **Mirror** | **Fluorochromes** |
| --- | --- | --- | --- | --- |
| Blue (488nm) | A | 710/50 | 685LP | PerCP-Cy5.5 |
|  | B | 525/50 | 505LP | FITC |
|  | C | 488/10 |  | SSC |
| Red (640nm) | A | 780/60 | 735LP | APC-Cy7 |
|  | B | 730/45 | 690LP | Alexa Fluor 700 |
|  | C | 670/30 |  | APC  Alexa Fluor 647 |
| Yellow-green (561nm) | A | 780/60 | 735LP | PE-Cy7 |
|  | B | 610/20 | 600LP | PE-Texas Red |
|  | C | 582/15 |  | PE |
| Violet (405nm) | A | 582/15 | 570LP | Aqua |
|  | B | 450/50 |  | eFluor 450 |
